# Acute stress response of the HPA-axis in children with Prader-Willi syndrome: new insights and consequences for clinical practice

**DOI:** 10.3389/fendo.2023.1146680

**Published:** 2023-05-23

**Authors:** Lionne N. Grootjen, Gerthe F. Kerkhof, Alicia F. Juriaans, Demi J. Trueba-Timmermans, Anita C. S. Hokken-Koelega

**Affiliations:** ^1^ Dutch Reference Center for Prader-Willi Syndrome, Rotterdam, Netherlands; ^2^ Department of Pediatrics, Subdivision of Endocrinology, Erasmus University Medical Center-Sophia Children’s Hospital, Rotterdam, Netherlands; ^3^ Dutch Growth Research Foundation, Rotterdam, Netherlands

**Keywords:** acute-stress response, central adrenal insufficiency, hypothalamic - pituitary - adrenal axis, children, Prader-Willi syndrome, hydrocortisone

## Abstract

**Background:**

Prader-Willi syndrome (PWS) is associated with hypothalamic dysfunction. It has been reported that the HPA axis might show a delayed response during acute stress, and it is unknown whether the response of the HPA-axis during acute stress changes with age in children with PWS.

**Aim:**

To investigate the HPA-axis response during an overnight single-dose metyrapone (MTP) test in children with PWS and to assess if the response changes with age, whether it is delayed and if it changes with repeated testing over time. In addition, we evaluated different cut-off points of ACTH and 11-DOC levels to assess stress-related central adrenal insufficiency (CAI).

**Methods:**

An overnight single-dose MTP test was performed in 93 children with PWS. Over time, 30 children had a second test and 11 children a third one. Children were divided into age groups (0-2 years, 2-4 years, 4-8 years and > 8 years).

**Results:**

Most children did not have their lowest cortisol level at 7.30h, but at 04.00h. Their ACTH and 11-DOC peaks appeared several hours later, suggesting a delayed response. When evaluated according to a subnormal ACTH peak (13-33 pmol/L) more children had an subnormal response compared to evaluation based on a subnormal 11-doc peak (< 200 nmol/L). The percentage of children with a subnormal ACTH response ranged from 22.2 to 70.0% between the age groups, while the percentage of a subnormal 11-DOC response ranged from 7.7 to 20.6%. When using the ACTH peak for diagnosing acute-stress-related CAI, differences between age groups and with repeated testing over time were found, whereas there was no age difference when using the 11-DOC peak.

**Conclusion:**

Early morning ACTH or 11-DOC levels are not appropriate to determine acute stress-related CAI in children with PWS, thus multiple measurements throughout the night are needed for an accurate interpretation. Our data suggest a delayed response of the HPA-axis during acute stress. Using the 11-DOC peak for the test interpretation is less age-dependent than the ACTH peak. Repeated testing of the HPA-axis over time is not required, unless clinically indicated.

## 1 Introduction

Prader-Willi syndrome (PWS) is a rare syndrome caused by the lack of expression of genes in the PWS region on the paternally derived chromosome 15, due to a paternal deletion, maternal uniparental disomy (mUPD), an imprinting center defect (ICD) or paternal chromosomal translocation (1, 2). Clinical findings characterizing PWS are abnormal body composition, muscular hypotonia, developmental delay, behavioral problems, hyperphagia, obesity, short stature (2), and endocrine problems, like hypothyroidism (3), growth hormone deficiency (4) and hypogonadism (5). In addition, dysregulation of body temperature and pain is also often found in PWS. Hypothalamic dysfunction is likely to be the cause of most of these symptoms.

The mortality rate in subjects with PWS is high (3% per year until the age of 30 years) (6). A large percentage of deaths in adults is associated with the complications of obesity, but children had mostly a mild viral illness or upper respiratory tract infection as the cause of death. Also, sudden, unexpected deaths are described (7–9). As small adrenal glands were found during autopsy in children with PWS after an unexpected death (10, 11), it has been suggested that acute stress-related central adrenal insufficiency (CAI) might be partly responsible for these unexplained deaths.

In literature, there is no agreement on the prevalence of CAI during acute stress in PWS (12). Several studies, in children and adults, using different test methods and definitions of CAI, have shown contradictory results. A previous study by our group reported a prevalence of acute stress-related CAI of 60% in 25 children with PWS (median age 9.7 years) during an overnight single-dose metyrapone (MTP) test, based on an adrenocorticotropic hormone (ACTH) peak cutoff of < 33 pmol/L, according to Steiner et al (13). Since then, the use of this ACTH level as a cutoff for the diagnosis of CAI has been questioned (14) and other cut-off points were used (15). Several studies using other methods to diagnose CAI, found a prevalence in PWS ranging from 0-15% (14, 16–22). However, most of these studies used a low-dose ACTH test, which is considered an appropriate method for diagnosing primary and secondary adrenal insufficiency, but less adequate for diagnosing tertiary adrenal insufficiency, originating from (partial) hypothalamic insufficiency (14, 23). Recently, a study in adults found a prevalence of CAI of 1.2% using the multiple-dose MTP test, but this test does not test the acute stress-related response of the HPA-axis (24). We previously established that children with PWS are able to produce adequate cortisol levels in daily life (13, 25) and do not suffer from CAI requiring daily hydrocortisone treatment. However, based on the unexpected sudden deaths of children with PWS, the question remained whether these children are able to generate a sufficiently fast response of the HPA-axis during severe acute stress. This can be tested with an overnight, single-dose MTP test or an insulin tolerance test (ITT), although the latter is not performed in our clinic anymore, because of the risk of severe side effects. Interestingly, one study in 36 children with PWS found a delayed response of cortisol during an insulin tolerance test (ITT) in 64% of them in comparison with healthy children (16), which indicated that the response of the HPA-axis during acute stress is delayed.

The clinical consequences of under- or over diagnosing acute stress-related CAI are significant. Underdiagnosing can lead to potentially life-threatening situations such as an adrenal crisis during acute stress, and over diagnosing can lead to overuse of hydrocortisone, which might lead to unnecessary side effects. It is, therefore, important to expand the knowledge about acute stress-related adrenal insufficiency in PWS and to create recommendations on the necessity, frequency and repeating the diagnostic procedures for CAI in PWS (12). It has been reported that there is a negative correlation between age and the HPA-axis (18, 21), which suggest that the response of the HPA-axis during acute stress could change with age.

We analyzed the ACTH, 11-deoxycortisol (11-DOC) and cortisol levels at different time-points during an overnight single-dose MTP test in 93 Dutch children with PWS and investigated whether the response to the single-dose MTP test changes with age, using two different cut-off points of acute stress-related CAI based on either the ACTH or 11-DOC peak. In addition, we investigated whether the response of ACTH and 11-DOC after a single-dose of MTP is normal or delayed in (young) children with PWS. With this study, we aimed to increase our insights in the response of the HPA axis during acute stress in children with PWS and whether this would have consequences for clinical practice. We hypothesized that the hormonal responses after a single-dose of MTP are different between children and adults with PWS because these changes are associated with age. In addition, we hypothesized that only few children with PWS have CAI requiring daily hydrocortisone treatment or an increased dose during mild to moderate stress, but that the response of the HPA-axis during severe acute stress as tested during the single-dose MTP test, would be delayed in children with PWS.

## 2 Materials and methods

### 2.1 Patients

All participants were diagnosed with PWS, confirmed by methylation analysis of the PWS region, and participated in the Dutch PWS Cohort study (26, 27).

### 2.2 Design

In this prospective study, all children underwent at least one single-dose MTP test during an overnight stay at the Pediatric Intensive Care Unit of the Erasmus Medical Center/Sophia Children’s Hospital (Rotterdam, The Netherlands). MTP tests were performed between March 2006 and June 2022. When a child was ill at the time of the MTP test, the test was not performed. All children visited the Dutch PWS Reference Center in Rotterdam and received multidisciplinary care by the PWS team in collaboration with pediatric endocrinologists and pediatricians in other Dutch hospitals. The Dutch PWS Cohort study was approved by Medical Ethics Committee of the Erasmus University Medical Center. Written informed consent was obtained from parents and children older than 12 years. Assent was obtained from children younger than 12 years. The study was conducted according to the guidelines of the Declaration of Helsinki II (28).

### 2.3 Overnight single-dose metyrapone test

Metyrapone was administered at 23.30h, in a dose of 30mg/kg (maximum dose of 3 g) at the Pediatric Intensive Care Unit of the Erasmus Medical Center/Sophia Children’s Hospital (Rotterdam, The Netherlands). The GH dose was given around 19.00-20.00h in the evening prior to the start of the MTP test, thus approximately 3-4 hours before the first blood draw. Metyrapone blocks the synthesis of cortisol by inhibiting 11-β-hydroxylase type 1, which converts 11-deoxycortisol to cortisol. The decline in plasma cortisol stimulates ACTH production, which causes 11-deoxycortisol to accumulate before the enzyme blockade. In four healthy adults, the maximal decrease in cortisol levels was found at 2 hours after oral administration of MTP and maintained for 7 hours post-dose (40mg/kg) (29). After oral administration, metyrapone is absorbed and eliminated rapidly from the circulation (elimination half-life ≈ 2h). Metyrapol (the active metabolite of metyrapone) has an elimination half-life of ≈ 4h (30). In other studies, serum levels of ACTH, 11-DOC and cortisol were only measured at 7.30h in the morning (31–33), but in our study, blood samples for the analysis of ACTH, 11-deoxycortisol and cortisol were obtained at 23.30h, 04.00h, 06.00h and 07.30h. The tubes for ACTH determination were immediately stored on ice after the blood draw. During the MTP test, blood pressure, heart rate and oxygen saturation were measured. After the fasting blood sampling at 07.30h, a single dose of 25mg hydrocortisone was orally administered. The MTP results were defined inconclusive if cortisol was not suppressed (cortisol < 200 nmol/L) during the metyrapone test (MTP) at any blood draw (04.00h, 06.00h, 07.30h). We used two measures to diagnose CAI. According to Steiner et al (32), a patient has CAI requiring daily hydrocortisone treatment, if the ACTH peak is < 13 pmol/L at all timepoints (04.00h, 06.00h, 07.30h) and a subnormal response, not requiring daily hydrocortisone treatment, if the ACTH peak is between 13 – 33 pmol/L, and normal if ACTH peak is > 33 at either 04.00h, 06.00h or 07.30h (32). Based on the 11-deoxycortisol levels, an 11-DOC peak lower than 200 nmol/L at all timepoints was defined as subnormal (34).

### 2.4 Assays

Tubes for adrenocorticotropic hormone (ACTH) determination were immediately stored on ice after the blood draw. Blood samples were measured in the Biochemical and Endocrine laboratories of the Erasmus University Medical Center, Rotterdam. ACTH and cortisol levels were analyzed with Siemens Immulite 2000XPi and 11-deoxycortisol (11-DOC) with UPLC-MSMS (Waters TQS, Etten-Leur, The Netherlands).

### 2.5 Statistics

Statistical analyses were performed using IBM SPSS Statistics 25. Categorical variables were reported using frequencies (N (%)) and continuous variables were expressed as median ± interquartile range (IQR), as not all variables had a normal distribution. Correlation between age and the results of the MTP test were analyzed with Spearman’s Rho. Differences between the different age-categories were analyzed by Kruskall-Wallis tests. Categorical data were compared by Chi-Square tests. The longitudinal changes in patients who were tested multiple times were analyzed with Wilxocon signed rank test, McNemar test, Friedman test or the Cochran’s Q test. Level of significance was set at a p-value of 0.05.

## 3 Results

We performed 166 tests, of which 13 (7.8%) had incomplete lab results. Of the 153 complete tests, 17 (10.9%) were inconclusive, due to inadequate suppression of the cortisol levels (<200 nmol/L) at 04.00h, 06.00h or 07.30h. In total, 136 tests were included in this study. In 93 children, we performed one test, in 30 children we also performed a second test, in 11, a third one and in 2, a fourth one.

### 3.1 Baseline characteristics

**Table 1** presents the baseline characteristics of the 93 children, including 58 (62.4%) boys. Of all children, 50 had a paternal deletion and 34 a mUPD. The median (IQR) age at the first MTP test was 0.97 (0.48; 4.81) years.

**Table 1:** Baseline characteristics.

Number	93
Male (%)	58 (62.4)
Genotype (%) Deletion mUPD Other	50 (53.8) 34 (36.6) 9 (9.6)
Age (years)	0.97 (0.48; 4.81)
Age at start GH (years)	0.70 (0.40; 1.91)

Data presented as n (%) or as median (IQR).

### 3.2 ACTH, 11-DOC and cortisol levels during the overnight singe-dose MTP test in various age groups

An inverse correlation between age and serum levels of ACTH was found at time point 06.00h (*r*=-0.246*, p*=0.004), but there was no correlation between age and 11-DOC levels at any time point. An inverse correlation was also found between age and cortisol levels at time points 04.00h, 06.00h and 07.30h (*r*=-0.346, -0.328 and -0.390, resp. (all *p-*values <0.001). Based on the correlation between age and the results of the MTP test, we decided to present our results per age group.

**Table 2** presents the results of the MTP test per age group. At baseline (23.30h), there were no differences between the age groups. At 06.00h, a difference in ACTH levels was found between the age groups, with the highest ACTH levels found in children aged 0-2 years. At 04.00h and 06.00h, children aged > 8 years had lower cortisol levels than children aged 0-2 years (p<0.001). At 07.30h, cortisol levels were still suppressed in children aged > 8 years, while in children aged 0-2 years, the cortisol levels had increased > 200 nmol/L. At none of the timepoints a difference in 11-DOC levels was found between the age groups.

**Table 2:** Results of the MTP test in children with PWS based on age groups.

	23.30h			04.00h			06.00h			07.30h		
	ACTH (pmol/L)	11-DOC (nmol/L)	Cortisol (nmol/L)	ACTH (pmol/L)	11-DOC (nmol/L)	Cortisol (nmol/L)	ACTH (pmol/L)	11-DOC (nmol/L)	Cortisol (nmol/L)	ACTH (pmol/L)	11-DOC (nmol/L)	Cortisol (nmol/L)
Age 0-2 year	4.0 (2.8; 6.2)	1.8 (0.6; 6.5)	131 (79; 263)	16.9 (11.3; 30.0)	236.2 (155.3; 277.6)	70 (40; 113)*	42.8 (25.0; 72.4) ***^#^***	299.0 (195.9; 343.0)	236 (117; 349)*	32.2 (13.8; 77.8)	269.4 (166.5; 345.0)	315 (196; 441)*
Age 2-4 year	3.0 (1.8; 4.4)	2.1 (0.5; 5.4)	196 (107; 361)	11.2 (6.6; 22.1)	181.9 (83.2; 242.8)	53 (28; 74)*	20.8 (15.6; 44.0) ***^#^***	223.1 (150.0; 307.0)	218 (97; 334)*	21.8 (9.7; 64.2)	234.2 (122.8; 276.8)	323 (214; 416)*
Age 4-8 year	3.7 (2.8; 5.1)	4.0 (0.9; 11.8)	209 (121; 329)	13.5 (8.2; 37.2)	199.0 (137.6; 277.8)	38 (14; 78)*	31.8 (20.2; 55.3) ***^#^***	266.0 (220.5; 343.5)	113 (72; 309)*	26.1 (11.0; 84.6)	281.0 (186.5; 377)	173 (91.3; 269.0)*
Age > 8 years	4.0 (3.1; 6.4)	1.1 (0.5; 6.3)	110 (49; 243)	19.2 (8.7; 48.4)	204 (143.3; 254.2)	14 (14; 29)*	28.6 (14.7; 78.1) ***^#^***	261.1 (187.0; 302.8)	43 (14; 90)*	40.8 (18.5; 80.6)	280 (219.2; 402.9)	81 (27; 154)*

Data presented as median (IQR), * p-value < 0.001, ^#^ p<0.05 for the difference between the age groups in ACTH, cortisol or 11-DOC levels at 04.00h/06.00h/07.30h.

**Figure 1:**
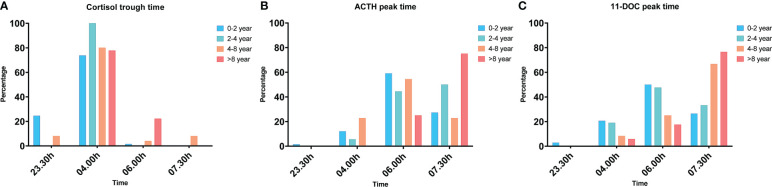
The percentage of children that had their cortisol trough time **(A)**, ACTH peak time **(B)** and 11-DOC peak time **(C)** per timepoint, in response to the overnight, single-dose MTP test, presented per age group. The cortisol trough time, ACTH peak time and 11-DOC peak time were significantly different between the age groups (p<0.001, p=0.025, p=0.007).

**Figure 1** presents the timing of the lowest cortisol levels and ACTH and 11-DOC peak levels per age group. The lowest cortisol levels were found at 04.00h in all age groups, however, 88.5% of children did not have their ACTH peak and 83.8% not their 11-DOC peak at the same time, indicating a delayed response of the HPA-axis after the MTP administration.

Seventy-five percent of the children aged > 8 years had their ACTH peak at 07.30h and 76% had their 11-DOC peak at 07.30h. Sixty percent of the children aged 0-2 years had their ACTH peak at 06.00h and 50% of them had their 11-DOC peak at 06.00h.

Timing of the peak ACTH and 11-DOC and lowest cortisol levels were neither significantly different between boys and girls nor between the different genetic subtypes (all p-values > 0.206)

### 3.3 The use of different cut-off points to diagnose subnormal acute stress-related response per age group

**Table 3** presents an overview of the subnormal acute stress-related response based on the different cut-off points of ACTH and 11-DOC peak levels after a single MTP administration per age group. Since most ACTH and 11-DOC peaks were not at 07.30h (as presented in **Table 2**; **Figure 1**), we investigated the ACTH and 11-DOC levels at all time points during the entire overnight MTP test. An abnormal ACTH peak (< 13 pmol/L) was not significantly different between the age groups as it was found in 1 only child. The prevalence of a subnormal ACTH peak (13 – 33 pmol/L) was different between the age groups (p=0.007), being 22.2% in children aged 0-2 years and 70.0% in children aged > 8 years. The prevalence of a subnormal 11-DOC peak (< 200 nmol/L) was 20.6% in children aged 0-2 years. 7.7% in those aged 4-8 years and 10.0% in those aged > 8 years (*p* = 0.641).

**Table 3:** ACTH and 11-DOC peak levels and per age group.

	0-2 year (n=63)	2-4 year (n=7)	4-8 year (n=13)	>8 years (n=10)	p-value
Peak ACTH < 13 pmol/L	1 (1.6)	0	0	0	0.923
Peak ACTH 13-33 pmol/L	14 (22.2)	2 (28.6)	7 (53.8)	7 (70.0)	0.007
Peak ACTH > 33 pmol/L	48 (76.2)	5 (71.4)	6 (46.2)	3 (30.0)	0.005
Peak 11-DOC <200 nmol/L	13 (20.6)	1 (14.3)	1 (7.7)	1 (10.0)	0.641

Data presented as n (%).

### 3.4 Results of repeated MTP tests over time

**Table 4** presents the results of the repeated MTP tests. Thirty children had 2 tests over time and 11 children had 3 tests. The prevalence of an abnormal ACTH peak < 13 pmol/L was very low and not different over time, but a higher prevalence of a subnormal ACTH peak between 13-33 pmol/L was found at the second test, when children were older. No differences in ACTH peak levels were found between the first, second and third test in 11 children with 3 MTP tests. The prevalence of an subnormal response based on a 11-DOC peak < 200 nmol/L (10-13%) did not change over time.

**Table 4:** Longitudinal changes in ACTH and 11-DOC peak levels.

	First MTP (n=30)	Second MTP (n=30)	p-value*	Third MTP (n=11)	p-value**
Age (years)	0.75 (0.37; 2.69)	3.05 (1.26; 5.66)	<0.001	5.55 (3.55; 9.35)	<0.001
Peak ACTH < 13 pmol/L	1 (3.3)	1 (3.3)	0.999	0	0.368
Peak ACTH 13-33 pmol/L	4 (13.3)	17 (56.7)	<0.001	4 (36.4)	0.325
Peak ACTH > 33 pmol/L	25 (83.3)	12 (40.0)	<0.001	7 (63.6)	0.197
Peak 11-DOC (<200 nmol/L)	4 (13.3)	3 (10.7)	0.999	1 (10.0)	0.368

Data presented as n (%).

*=p-value for the difference between first and second MTP test.

**=p-value for the difference between all three tests (for n=11).

At the first test, 18 children were still prior to the start of GH treatment, but all children started GH before the second test. A 11-DOC peak < 200 nmol/L was found in one of these eighteen patients at the first MTP test and in three patients at the second MTP test (p=0.625). An ACTH peak of < 13 nmol/L was found in none of the patients at the first test and in one patient at the second MTP test (p=0.999).

## 4 Discussion

This is the largest study to date investigating acute stress-related CAI in young children with PWS with the overnight single-dose MTP test. Most children had their lowest cortisol levels at 04.00h, but their ACTH and 11-DOC peak levels appeared several hours later at 06.00h or 07.30h, which might indicate a delayed response of the hypothalamic–pituitary–adrenal axis to acute stress in children with PWS. As most (young) children with PWS did not have their ACTH and 11-DOC peak levels and lowest cortisol levels at 07.30h, multiple measurements during the overnight, single-dose MTP test appear to be needed in children with PWS for an adequate interpretation of the HPA-axis response to a single-dose of MTP. The prevalence of a subnormal ACTH peak (13 - 33 pmol/L) during the overnight single-dose MTP was different between the age groups (ranged from 22.2 to 70.0%), but the prevalence of a subnormal 11-DOC peak (< 200 nmol/L) did not significantly change with age (ranged from 7.7 to 20.6%). In addition, the prevalence of a subnormal stress response based on the 11-DOC peak did not change with repeated MTP testing over time. We, therefore, conclude that the 11-DOC peak provides more consistent information than the ACTH peak to interpret the single-dose MTP test. We also showed that repeated MTP testing over time provides similar results.

Our study gives insight in the prevalence of acute stress-related CAI in children with PWS. Steiner et al. showed that an ACTH plasma level < 33 pmol/L as well as 11-DOC levels < 200 pmol/L are cut-off points with a high sensitivity of diagnosing CAI (32). However, they also showed that patients with an ACTH peak level between 13-33 pmol/L did not require daily substitution with hydrocortisone treatment and mentioned that an ACTH peak level < 33 pmol/L could lead to overdiagnosis of CAI. They concluded that plasma ACTH levels of 13 pmol/L would probably be sufficient to produce cortisol levels within the normal range (32). ACTH levels between 13-33 pmol/L, however, indicated a subnormal response during the MTP test, not requiring daily hydrocortisone treatment (32), but this would not exclude the need for hydrocortisone medication during stressful events. We investigated all three ACTH cut-off levels in the present study. Younger children (aged 0-2 years) were more likely to have a normal ACTH peak compared to older children, who were more likely to have a subnormal ACTH peak after MTP, while no correlation between age and 11-DOC was found. Although the prevalence of a subnormal ACTH peak changed with age, the prevalence of a 11-DOC peak < 200 nmol/L did not change with age, showing that a sufficient 11-DOC peak was reached even with a lower ACTH peak level in the older children. This might suggest that the response of the hypothalamic-pituitary-adrenal (HPA) axis improves with age and that the adrenal gland becomes more sensitive to ACTH during childhood. However, further research is needed to gain more insight in the potential changes in the HPA-axis during puberty. We found that the prevalence of a subnormal 11-DOC peak < 200 nmol/L decreased from 22.2% in children aged 0-2 years to 11.1% in children aged > 8 years, albeit not significantly. We, therefore, conclude that using the cut-off point of 11-DOC peak < 200 nmol/L is more consistent and probably more appropriate than using the ACTH peak as cut-off point.

In other studies, serum levels of ACTH, 11-DOC and cortisol were only measured at 7.30h in the morning (31–33) and the cut-off point of 11-DOC for diagnosing CAI in adults is only based on the measurement at 07.30h (32, 34). We found that the majority of the very young children had their ACTH and 11-DOC peaks at 06.00h. If only one measurement at 07.30h would have been performed, many young children would have been wrongly diagnosed. In addition, several tests would have been issued as inconclusive, as most very young children had cortisol levels > 200 nmol/L at 07.30h, while their cortisol was sufficiently suppressed at an earlier time point, mostly at 04.00h. Therefore, multiple measurements during the overnight, single-dose MTP test are needed for an accurate interpretation of the test in young children with PWS.

Almost all children had their lowest cortisol level measured at 04.00h, but almost none of them had their ACTH or 11-DOC peak at that same time. Metyrapone is quickly absorbed after oral administration (30), and maximal suppression of cortisol is found after 2 hours (29). In times of acute stress, the amygdala activates the HPA axis by signaling the hypothalamus to release CRH (35), which triggers the release of ACTH from the anterior pituitary, which in turn stimulates the adrenal cortex to release cortisol (36). This is a fast process, as approximately 15 minutes after the onset of stress, cortisol levels are rising (37, 38). We would, therefore, have expected to find high levels of ACTH and 11-DOC at 04.00h, at 4.5 hours after the single-dose MTP. Our results are in line with the findings of Oto et al., who found a delayed peak in ACTH and cortisol levels during an insulin tolerance test (ITT) in children with PWS compared to healthy children (16). Together, these findings might indicate that there is a delayed response of ACTH and 11-DOC in children with PWS during severe acute stress, when a fast response of ACTH is required. It might also explain the difference in results of our previous study (13) compared to the study performed in adults (24). In that study, the 24-hour multiple dose MTP test was used, leading to a much longer period for the HPA axis to respond with adequate levels of 11-DOC at 24 hours after the start. We have previously established that most children with PWS are able to produce adequate cortisol levels in daily life (13, 25), thus not requiring treatment with hydrocortisone in everyday life. But a delayed response of the HPA-axis during acute stress, as found by Oto et al. and suggested by our study, can result in symptoms similar to acute stress-related CAI. Although this delayed response of the HPA axis would formally not be marked as an insufficient result of the MTP test, treatment during severe acute stress may be needed.

Notably, although all children included in our study had at least one cortisol measurement < 200 nmol/L, the early morning cortisol levels were higher, especially in the very young children. According to the interpretation of the MTP test in adults, results are inconclusive when cortisol levels are not suppressed at 07.30h. We measured the hormone levels multiple times during the night, to provide more insight in the suppression of cortisol levels and the peak ACTH and 11-DOC levels. We found that cortisol levels were lowest at 04.00h in almost all children. Interestingly, although all children had a sufficient suppression of cortisol after the single-dose of metyrapone, cortisol levels increased again after 04.00h in the very young children, which resulted in cortisol levels > 200 nmol/L at 07.30h. It might be that the very young children have a faster metabolism of metyrapone and therefore a faster elimination of metyrapone and metyrapol, and that the HPA axis is already restored in the early morning. Another explanation might be that the dose of 30mg/kg is too low for very young children to maintain a sufficiently long blockade of 11-β-hydroxylase type 1. Current dosing is calculated as mg/kg, which results in a relatively low dose in very young children (with a small body size), compared to older children. For example, a young child of 5 kg with a body surface area (BSA) of 0.3 m2 receives 150 mg metyrapone, which corresponds to 500 mg/m2, while an older child of 50 kg with a BSA of 1.6 m2 receives 1500 mg metyrapone, corresponding with a much higher dose of 938 mg/m2. Dosing metyrapone in mg/m2 might solve this. However, this needs to be investigated further and the dose should not increase the maximum of 3000mg. As most infants with PWS were tube fed and received the MTP *via* the tube, these very young children had a guaranteed intake of MTP. We recommend to perform multiple measurements during the night, when the overnight single-dose MTP test is performed to study acute stress-related CAI in young children, as the HPA axis might be restored in the early morning in young children with PWS.

This is the first study describing the results of repeated overnight single-dose MTP tests over time in children with PWS. As a negative correlation between age and peak cortisol levels to the low dose ACTH test was previously described (18, 21), there was a need for investigating changes in the function of the HPA axis over time. Our results show no difference over time in the prevalence of an insufficient 11-DOC peak level. Repeating the MTP tests over time is, therefore, not required, unless there is a clinical indication.

There are, to our knowledge, no overnight, singe-dose MTP test results reported in healthy young children and thus no reference values of peak ACTH and 11-DOC levels for the pediatric population. Although our results suggest a delayed response of the HPA-axis during acute stress, in line with Oto et al. (16), we could not prove this hypothesis due to the lack of an adequate control group. The use of age-matched healthy children as a control group would have been the first-choice to compare the response of the HPA-axis in (young) children with PWS. However, it would have been unethical to perform the MTP test in healthy children, since the test is invasive and requires overnight admission at the paediatric ICU because of a potential risk of an adrenal crisis during the night. Since data about the MTP test in children are very scarce, we used the published cut-off levels for the test in healthy adults. As there are hormonal differences between children, adolescents and adults, our results should interpreted with caution. However, all studies describing the results of the overnight single-dose MTP test in adults have only drawn blood at 07.30h, so it is unknown how ACTH and 11-DOC levels changed during the night. Although our study provides more insight into the HPA axis response to a single-dose of MTP as a proxy for acute stress in children with PWS, further research, also on the cut-off levels in healthy children and adolescents, is warranted.

In our previous study, the prevalence of acute stress-related CAI was 60% (13). This prevalence was based on an ACTH peak < 33 pmol/L as the cut-off, recommended by Steiner et al (32). As it might be that the use of a cut-off point of ACTH < 33 pmol/L leads to overdiagnosis of stress-related CAI, we decided to differentiate between an abnormal ACTH response (< 13 pmol/L at all timepoints) requiring daily hydrocortisone treatment and subnormal (ACTH response of 13-33 pmol/L), not requiring daily hydrocortisone treatment and also evaluated a cut-off point of 11-DOC < 200 nmol/L. In the present study, we found only 1 child with an abnormal response of ACTH, but a subnormal response was found in a higher percentage of children. Over the years, we observed a decline in the need for hydrocortisone stress-dose medication during mild to moderate illness in the children followed in our PWS Reference Center. Nonetheless, it remains important to be aware that acute stress-related CAI may be part of the PWS phenotype during childhood, due to a delayed acute stress response of ACTH.

Untreated growth hormone deficiency may mask symptoms of CAI, as low serum insulin-like growth factor-1 levels lead to higher levels of the enzyme that converts cortisone to cortisol (39). In 18 patients who were prior to the start of GH, we repeated the MTP tests during GH treatment and found no change in the prevalence of acute stress-related CAI. Other studies in PWS also found no difference between GH-treated patients and non-GH treated patients (14, 24). These findings suggest that GH treatment in children with PWS has no influence on the HPA-axis.

In conclusion, sing 11-DOC for the interpretation of the MTP test seems more accurate and less age-dependent than ACTH peak levels. The percentage of a subnormal ACTH response ranged from 22.2 to 70.0% between the age groups and the percentage of a subnormal 11-DOC response from 7.7 to 20.6%. Multiple laboratory measurements during the night after MTP administration are needed, as most younger children had their lowest cortisol level at 04.00h and their ACTH and 11-DOC peak at 06.00h. epeated testing for CAI should not be standard procedure, but should only be performed when there is a clinical suspicion of CAI. The increase in ACTH and 11-DOC during MTP test was found several hours after the lowest cortisol levels, suggesting a delayed response of ACTH during the overnight MTP test. As this delayed response of ACTH might lead to symptoms of CAI during acute stress, it seems safer to treat children with PWS with hydrocortisone during surgical procedures and severe acute illness (for example when the child is admitted to the Intensive Care Unit), while standard use of hydrocortisone during mild to moderate stressful conditions in daily life might not be indicated.

## Data availability statement

The raw data supporting the conclusions of this article will be made available by the authors, without undue reservation.

## Ethics statement

The studies involving human participants were reviewed and approved by Medical Ethics Review Committee of the Erasmus MC. Written informed consent to participate in this study was provided by the participants’ legal guardian/next of kin.

## Author contributions

LG and AH-K were responsible for conceptualization, analysis was performed by LG, GK and AH-K were responsible for the methodology, supervision was done by GK and AH-K. Writing – original draft performed by LG, review and editing performed by LG, GK, AJ, DT-T AND AH-K. All authors contributed to the article and approved the submitted version.
